# Screening for inflammation and treatment with colchicine in adults with known cardiovascular disease: an analysis of 13.5 million patients

**DOI:** 10.3389/fmed.2026.1882786

**Published:** 2026-09-10

**Authors:** Arch G. Mainous, Lu Yin, Frank A. Orlando

**Affiliations:** Department of Community Health and Family Medicine, University of Florida, Gainesville, FL, United States

**Keywords:** clinical guideline, clinical guideline adherence, colchicine, inflammation, screening

## Abstract

**Background:**

Identifying and treating inflammation is a recommended atherosclerotic cardiovascular disease (ASCVD) prevention strategy. The practice of screening for and treating inflammation is unknown. This study examined screening for inflammation and treatment with colchicine among patients with established ASCVD.

**Methods:**

Using the Epic COSMOS electronic health record (EHR) database, we compared adults with known ASCVD between two time frames: (1) 2023 (colchicine FDA approved for ASCVD prevention) until April 2026 and (2) September 2025 [American College of Cardiology (ACC) scientific statement released] until April 2026. We examined screening for inflammation and treatment of inflammation. Chi-square tests compared differences before and after the ACC scientific statement on inflammation.

**Results:**

The study contained 13.5 million adults aged 18 years or older diagnosed within the EHR with ASCVD. Only 1.2% of the patients with ASCVD were on colchicine. The proportion of patients with known ASCVD who were screened to assess inflammation was also very low (3.1%). Among patients who had hs-CRP measurements, 65.3% had elevated levels. Additionally, even in light of documented inflammation, the proportion of patients with elevated documented inflammation are on colchicine was low but did increase after the ACC scientific statement from 2.1% to 2.9%.

**Conclusion:**

The evidence on inflammation and its role in ASCVD is substantial. Screening for inflammation and treatment of the inflammation pathway in patients with ASCVD is very low and not consistent with evidence-based practice.

## Introduction

Inflammation is a recognized key component of atherosclerotic cardiovascular disease (ASCVD) with several decades of evidence (1). Common markers of inflammation such as C-reactive protein (CRP), have been proven to be reliable and important markers of cardiovascular disease risk. In 2002, the Centers for Disease Control and Prevention and the American Heart Association convened the panel “CDC/AHA Workshop on Inflammatory Markers and Cardiovascular Disease: Applications to Clinical and Public Health Practice” (2). In the subsequent 24 years, even more evidence has accumulated focusing on the role of inflammation in the pathogenesis of ASCVD and the corresponding utility of high-sensitivity CRP (hs-CRP) and preventive treatments, particularly for patients with known ASCVD.

Large trials like the JUPITER trial supported the inflammatory hypothesis in ASCVD (3). It demonstrated that statin therapy reduces by 50% the risk of heart attack and stroke among men and women with low levels of low-density lipoprotein (LDL)-cholesterol but elevated levels of CRP. Subsequent trials like CANTOS and CIRT were proof-of-concept studies focused on specific treatment strategies to lower inflammation and decrease the likelihood of ASCVD events (4–6).

Recently, interest has focused on colchicine, a drug with significant anti-inflammatory properties, as a strategy to impact the inflammation pathway and improve ASCVD outcomes. Colchicine was particularly safe and effective in trials for a secondary prevention strategy in patients with established ASCVD irrespective of hs-CRP levels (7–9). Colchicine, marketed as Lodoco, has since become the first drug approved by the US Food and Drug Administration (FDA) for ASCVD prevention by focusing on the anti-inflammatory pathway (10). Prescribing colchicine for secondary ASCVD prevention necessitates consideration to drug–drug interactions, cytopenias, neuromuscular toxicity, gastrointestinal intolerance, and renal or hepatic impairment (7–9, 11, 12). In 2025, the American College of Cardiology (ACC) scientific statement pointed out that the evidence linking inflammation with ASCVD is strong and clinically actionable (13). However, the integration of this evidence-based information into clinical practice is unknown.

The purpose of this study was to examine the general use of testing for inflammation with hs-CRP and use of colchicine among patients with known ASCVD. We investigated this in normal clinical practice in a nationwide electronic medical record registry containing millions of US patients in the time frame since colchicine was approved as a treatment for inflammation in patients with known ASCVD and in the later time period since the ACC encouraged inflammation as a treatment target to prevent ASCVD events.

## Methods

### Database

We analyzed the COSMOS database. COSMOS is based on the Epic electronic health record (EHR) which is used throughout health systems in the US and contains data on more than 300 million patients. COSMOS integrates both inpatient and outpatient charts into a single, comprehensive record. This includes drugs and encompasses patients who move between health systems, if the new health system also uses the Epic EHR.

### Time frame under study

The time frame examined was from 2023, the year colchicine was FDA approved to treat inflammation among patients with known ASCVD to prevent future ASCVD events, until April 2026. A second time frame was also examined, from September 2025 until April 2026. The ACC scientific statement on assessing inflammation via hs-CRP and treating inflammation was released in September 2025.

### Population under study

The population under study are adult patients aged 18 and older with established or known ASCVD. ASCVD was defined according to the American Heart Association as acute coronary syndromes, myocardial infarction, stable or unstable angina, arterial revascularization, stroke, transient ischemic attack, or peripheral arterial disease of atherosclerotic origin (14). These are patients identified within the EHR as having had a myocardial infarction (ICD-10 codes I21, I22, I23) or history of myocardial infarction (ICD-10 code I25.2); stable or unstable angina due to ASCVD (I25.11x, I25.7x); arterial revascularization of coronary (ICD-10 codes Z95.1, Z95.5, Z98.61), peripheral (ICD-10 codes Z95.820, Z98.62), or other vascular (ICD-10 code Z95.828); stroke (ICD-10 codes I63), history of stroke (ICD-10 code Z86.73), or late effect stroke symptoms (ICD-10 code I69); transient ischemic attack (ICD-10 code G45.x); and peripheral artery disease of atherosclerotic origin (ICD-10 codes I70.21x–I70.26x, I74, I70.3x–I70.7x).

Because patients could be in the EHR database multiple times, once they were identified as having ASCVD we used the most recent data on the patient to document their care.

### hs-CRP and colchicine

In patients with known ASCVD, hs-CRP levels above 3.0 mg/L were considered elevated and used to indicate inflammation (2).

Low-dose colchicine for secondary prevention of ASCVD is prescribed once daily. Patients were selected if they were using generic colchicine (0.6 mg) or the brand FDA approved for secondary ASCVD prevention (Lodoco 0.5 mg) once daily for at least 30 days. This helped to distinguish between colchicine that may have been prescribed for an acute gout flare, a common comorbidity in patients with established ASCVD. Since gout flares are an acute inflammatory episode requiring multiple doses of colchicine over 1 h, a once daily use for at least 30 days would limit colchicine use to individuals on a preventive medicine for the inflammation pathway of ASCVD.

### Analysis

The initial analysis focused on the number of patients with known ASCVD who received an hs-CRP test for inflammation. For those patients who received an hs-CRP test, we categorized the patients as either having an elevated hs-CRP or not. Among those with known ASCVD, we computed how many patients were prescribed colchicine stratified by elevated hs-CRP. This pathway was computed for the time from 2023 to September 2025, a time consistent with the approval of colchicine for ASCVD treatment but before the ACC scientific statement on inflammation identification and treatment and a comparison group from September 2025 to April 2026, a time after the ACC scientific recommendation. Chi-square tests were conducted to compare the differences before and after the ACC scientific statement on inflammation. The study used a level of *p* < 0.05 to indicate statistical significance.

Additionally, we computed the patient characteristics that distinguished patients who were tested for inflammation and those who were not and patients who were prescribed colchicine and did not. Patient characteristics included age, sex, race, insurance. Even though all of the patients had known ASCVD and would therefore be candidates for colchicine, in an effort to overcome what may be possible misclassification of colchicine use for a condition other than for ASCVD prevention we classified the study population into two subgroups: (1) a confounder group, consisting of patients with comorbid conditions for which colchicine is an indicated treatment, and (2) a group of patients without colchicine indications. The comorbidity conditions that could be considered eligible for colchicine were Gout (M10.x); Pericarditis (I30.x); Familial Mediterranean fever (FMF) (E85.0); and Behcet disease (M35.2). Among patients with the comorbid conditions, 94.4% had Gout, 5.1% had Pericarditis, 0.5% had Behcet's disease, and only a negligible number had familial Mediterranean fever (FMF). All the baseline comorbidities were ascertained prior to the date of colchicine prescription. A comorbidity was considered present if its recorded diagnosis date occurred before the colchicine prescription date. Diagnoses first recorded on or after the index date were not considered baseline comorbidities. We also performed a multivariable logistic regression analysis to examine factors associated with colchicine use. The outcome variable was receipt of colchicine, and the model adjusted for insurance status, age, sex, and the comorbidity groups described above.

Our study was approved by the University of Florida Institutional Review Board as nonhuman research (Protocol # NH00054117).

## Results

The population characteristics of adults aged 18 years or older and diagnosed with ASCVD are shown in Table 1. There were 13.5 million people in the study. Table 2 presents the overall percentages and the percentages before and after before and after the ACC scientific statement on inflammation across the four study groups. The overall utilization of colchicine was low, although an increase in colchicine use was observed following the ACC scientific statement. All comparisons were statistically significant (*p* < 0.001).

**Table 1:** Population estimates for demographic characteristics of adults aged ≥18 years and diagnosed with ASCVD (*N* = 13.5 million).

Factors	Population proportion (%)
Sex (%)	
Male	53.4
Female	46.6
Age (%)	
18–39 years	3.9
40–59 years	18.2
60–79 years	53.4
80 and above	24.5
Insurance (%)	
Self-pay	1.4
Insured	98.6
Race (%)	
Non-Hispanic White	61.4
Non-Hispanic Black	12.8
Hispanic	3.1
Other	22.7
Colchicine indication status (%)^a^	
Patients without colchicine indications	97.6
Patients with colchicine indications	2.4

a The conditions that could be considered eligible for colchicine were Gout; Pericarditis; Familial Mediterranean fever; and Behcet disease.

**Table 2:** Impact of the ACC scientific statement on identifying inflammation and treating inflammation with colchicine in patients with known ASCVD.

	Overall (*N* = 13,517,566)	Before the ACC statement but after colchicine was FDA approved (*N* = 7,487,600)		After the ACC statement (*N* = 6,029,966)		*p*
		0.6 mg	0.5 mg	0.6 mg	0.5 mg	
Proportion of patients with known ASCVD taking colchicine to control inflammation	1.2% (*N* = 163,774)	1.1% (*N* = 78,704)	0.003% (*N* = 193)	1.4% (*N* = 84,620)	0.004% (*N* = 257)	<0.001
Proportion of patients with known ASCVD screening hs-CRP to assess inflammation	3.1% (*N* = 415,190)	2.7% (*N* = 201,734)		3.5% (*N* = 213,456)		<0.001
Proportion of patients with hs-CRP measurements who have elevated levels	65.3% (*N* = 271,316)	69.2% (*N* = 139,555)		61.7% (*N* = 131,761)		<0.001
Proportion of patients with elevated hs-CRP on colchicine	2.5% (*N* = 6,840)	2.1% (*N* = 2,932)	0.016% (*N* = 23)	2.9% (*N* = 3,842)	0.033% (*N* = 43)	<0.001

Only 2.4% of the population had one of the confounders. Among everyone (patients with colchicine indications or patients without colchicine indications) 1.2% were prescribed colchicine. Among the “patients without colchicine indications” 0.3% were prescribed colchicine. Among the “patients with colchicine indications” 16.9% were prescribed colchicine.

In a logistic regression, after adjustment for age, sex, insurance status, and comorbidities, patients without comorbidities had substantially lower odds of receiving a colchicine prescription (OR 0.015, 95% CI 0.0148–0.0152). These findings indicate that colchicine utilization among patients with ASCVD remains low, although use is relatively higher among patients with comorbidities. The full regression model with the values of the control variables is presented in a Supplementary Table.

## Discussion

Inflammation has long been known to play a critical role in ASCVD progression, morbidity, and mortality (1, 2). The recent ACC scientific statement and the FDA approval of colchicine for secondary prevention of ASCVD by focusing on the inflammatory pathway have provided clinicians with guidance on both identifying inflammation and treatment (13). The results of this study of 13.5 million patients with known ASCVD seen in US clinical practice indicates that although there was a statistically significant impact of the scientific statement on practice, the clinical significance was limited. The proportion of patients identified as having inflammation, defined as elevated hs-CRP, and on colchicine to treat inflammation increased from 2.1% to only 2.9%. Clearly, identifying and treating inflammation as secondary ASCVD prevention is very uncommon and has definite room for improvement.

The current findings indicate that these evidence-based recommendations have not been integrated into the care for patients with known ASCVD. It is unclear if this is due to a lack of knowledge or attitudes toward the efficacy of identifying and treating inflammation for ASCVD morbidity and mortality prevention. The general knowledge of the role of inflammation in cardiovascular disease suggests the need to accelerate the adoption of evidence-based, guideline-directed anti-inflammatory therapy through focused dissemination and implementation research. Perhaps the integration into practice could be helped if inflammation was to be embraced as a quality measure with corresponding financial implications.

hs-CRP is a validated inflammatory biomarker and guideline-recommended risk-enhancing factor for use in ASCVD risk stratification, and reflects persistent, residual inflammation in ASCVD (15, 16). On the other hand, standard CRP is the assay used in clinical practice to evaluate for acute inflammation and disease activity in infections like osteomyelitis and auto-immune disorders like rheumatoid arthritis (RA) (17, 18). The two tests measure the same protein but differ in sensitivity and the clinical range they are designed to detect. For example, low-grade systemic inflammation detected by hs-CRP is neither a predictor of incident cancer nor of the risk of developing major systemic inflammatory disorders, such as RA or inflammatory bowel disease (19). Furthermore, since CRP is an acute phase reactant that increases transiently during major trauma and infection, hs-CRP screening should generally be avoided in practice during acute clinical events like these (13). However, merely having a diagnosis of an inflammatory, autoimmune disorder like RA does not preclude screening for chronic low-grade inflammation using hs-CRP per the ACC statement. Persistently elevated hs-CRP levels exist since many individuals with chronic inflammatory, autoimmune disorders also suffer from premature ASCVD (13).

The objective of the study was to determine how often clinicians order hs-CRP and prescribe colchicine in patients with known ASCVD, and since the population is already limited to patients with ASCVD, the design would not represent the real world if ASCVD patients were removed for having an RA diagnosis or because their hs-CRP was >10 mg/L. Having an hs-CRP >10 mg/L does not preclude preventative treatment for ASCVD using colchicine per the same statement, and while patients with osteomyelitis or RA are not treated with colchicine, the entire study population of patients with known ASCVD qualified for treatment with colchicine. Per the ACC statement, hs-CRP >10 mg/L may reflect a transient infection or other acute-phase response and should therefore be repeated in 2–3 weeks with the lower value used for risk prediction (13).

Adoption of colchicine fits within the broader area of secondary ASCVD prevention that includes lifestyle therapies, antiplatelet therapy, and risk factor management such as cholesterol, high blood pressure, and diabetes (15, 20, 21). Cost-effectiveness analyses of the COLCOT trial have shown that adding low-dose colchicine (0.5 mg daily) to standard-of-care therapy after myocardial infarction is an economically dominant strategy (22). Despite this cost savings, insurance coverage for Lodoco is variable and often restricted, requiring various hurdles like prior authorizations and step therapy (23). Hence, in real-world practice, prescribing generic colchicine for secondary ASCVD prevention is frequently easier and necessary for plans requiring step therapy.

More recent evidence from the CLEAR trial reported neutral findings on composite cardiovascular outcomes when colchicine was started in the setting of acute myocardial infarction within 72 h of percutaneous coronary intervention (24). The potential impact of these recent results on colchicine prescribing practices may not be fully reflected in the present analysis, as changes in clinical practice often occur gradually following the publication of new evidence. It is important to note, however, that this population was different than that of the original COLCOT trial which had positive results when colchicine was started within 30 days following myocardial infarction once patients had been stabilized (8). Furthermore, a recent meta-analysis that included both CLEAR and COLCOT trials showed that Colchicine reduced the risk of non-fatal ischaemic events in patients with established ASCVD, calling for future studies to identify populations who benefit the most from colchcine (25). We found one recent, real-world study that evaluated a population of patients with stable ASCVD taking a statin to identify the number of new colchicine users following the publication of the COLCOT and LoDoCo2 trials in 2019–2020 (26). While this study did not measure colchcine use since the later time period of the 2025 ACC recommendation like the current study and only evaluated up to 170,000 patients from a single health system, the results of that study were similar to the current study. They revealed a gap between published evidence and clinical implementation as colchicine use for ASCVD prevention remained very low (<1%) among eligible patients despite a modest uptake increase after COLCOT and LoDoCo2 publication (26). Using the National Health and Nutrition Examination Survey, it was estimated that broader implementation of colchicine to standard cardiovascular management in eligible populations could potentially prevent 226,000 major adverse cardiovascular events in eligible populations (26).

There are several limitations to this study. First, even though our definition of colchicine used for ASCVD inflammation was logical for both dose and duration and unlikely to be one used for other conditions, it may have some misclassification bias with other conditions. In the EHR data of COSMOS, drug prescriptions do not indicate the diagnosis for which they were prescribed. While colchicine is most commonly used short-term for acute gout flares, daily use can also be used for gout prophylaxis. However, daily colchicine for gout prevention is much less common than daily allopurinol, especially amongst a population with established ASCVD on a statin. Another less common indication for daily colchicine use is pericarditis, but these patients typically take colchicine twice per day. Second, we attempted to be as encompassing as possible to clinician behavior realizing that for patient cost reasons, some clinicians prescribe generics similar to name brand drugs. Low-dose colchicine consistent with the ACC statement on ASCVD prevention currently only comes in the 0.5 mg tablet as the brand Lodoco, which may lack coverage by some insurances and therefore not be affordable for every patient. We evaluated all patients on daily colchicine for 30 days irrespective of dosage. We therefore included patients on 0.6 mg colchicine daily to include patients who were using this dosage off-label for secondary ASCVD prevention. Third, we used the standard and recommended measure for chronic low-grade systemic inflammation in ASCVD, hs-CRP (13). In the EHR data of COSMOS, laboratory data do not indicate the diagnosis for which they were ordered. As with the above limitation regarding diagnoses not being directly linked with prescribed drugs, the same issue with laboratory tests could play a significant role in interpreting test motivation if testing itself was very common, but any misclassification in a situation like the current study where the rate of testing is very low only reinforces the lack of adherence to the scientific statement. More importantly, although elevated hs-CRP could indicate an acute illness, therefore misclassifying some patients and rationale for the test, an hs-CRP >10.0 mg/L does not always indicate acute illness. Physicians could still choose to use colchicine in these patients to reduce ASCVD risk. Moreover, there may be differences in individual clinical eligibility for colchicine, which may be influenced by renal or hepatic dysfunction, drug interactions, intolerance, comorbidities, and other clinical considerations which may be missed in this EHR analysis. Fourth, the design cannot ensure that observed changes in hs-CRP testing or colchicine prescribing is caused by the ACC statement, and the examined 8-month post-ACC statement period is relatively short for adoption of a scientific statement. However, evaluations of national guidelines and scientific recommendations have used similar designs (27, 28), innovators and early adopters make up 16% of an implementation population (29), and Lodoco has been FDA approved for use for the examined indication of since 2023 (30).

## Conclusion

The evidence on inflammation and its role in ASCVD is substantial. This study found that even though the American College of Cardiology scientific statement recommends screening for inflammation and treatment of the inflammation pathway in patients with ASCVD, the practice is very uncommon. Only 1.2% of the patients with ASCVD were on the FDA approved and recommended anti-inflammatory medicine colchicine. Although the analysis of this real-world investigation using EHR data gives a view to actual clinical practice, causality cannot be concluded from the data. However, clinician adoption of strategies to identify and treat inflammation has a long way to go with most eligible patients missing out on evidence-based care. With new anti-inflammatory treatments like colchicine, it is time to incorporate evidence-based practice to make inflammation a much more primary treatment target.

## Data Availability

Publicly available datasets were analyzed in this study. This data can be found here: Epic COSMOS.
