# Supplementary material for: Screening for inflammation and treatment with colchicine in adults with known cardiovascular disease: an analysis of 13.5 million patients
Source: Front Med (Lausanne). 2026 Sep 10;13:1882786. doi: 10.3389/fmed.2026.1882786 (PMC13600809; doi:10.3389/fmed.2026.1882786)
Supplement: Supplementary file 1 [file Datasheet1.pdf]

Supplementary File

Full model adjusted logistic regression results examining receipt of colchicine.

	OR	CI(2.5%,97.5%)	P
(intercept)	0.41	0.40,0.43	<0.001
Patients without colchicine indications	0.01	0.01,0.01	<0.001
Age(40-59)	0.93	0.89,0.96	<0.001
Age(60-79)	0.98	0.95,1.01	0.23
Age(80+)	0.95	0.92,0.98	0.005
Male	1.14	1.12,1.15	<0.001
Self-pay	0.78	0.73,0.82	<0.001
